# Computational design of experimentally validated multi-epitopes vaccine against hepatitis E virus: An immunological approach

**DOI:** 10.1371/journal.pone.0294663

**Published:** 2023-12-14

**Authors:** Tasneem Anwar, Saba Ismail, Fahed Parvaiz, Sumra Wajid Abbasi, Fahad A. Al-Abbasi, Amira M. Alghamdi, Khalid Al-Regaiey, Asad Ul-Haq, Imdad Kaleem, Shahid Bashir, Yasir Waheed

**Affiliations:** 1 Department of Biosciences, COMSATS University Islamabad (CUI), Islamabad, Pakistan; 2 Department of Pharmacy and Pharmaceutical Sciences, University of Alberta, Edmonton, Alberta, Canada; 3 Department of Biological Sciences, National University of Medical Sciences, Rawalpindi, Pakistan; 4 Department of Biochemistry, Faculty of Sciences, King Abdulaziz University, Jeddah, Saudi Arabia; 5 Department of Physiology, King Saud University, Riyadh, Saudi Arabia; 6 Division of Rheumatology, Department of Internal Medicine, Soonchunhyang University Seoul Hospital, Seoul, Republic of Korea; 7 Neuroscience Center, King Fahad Specialist Hospital Dammam, Dammam, Saudi Arabia; 8 Office of Research, Innovation & Commercialization, Shaheed Zulfiqar Ali Bhutto Medical University (SZABMU), Islamabad, Pakistan; 9 Gilbert and Rose-Marie Chagoury School of Medicine, Lebanese American University, Byblos, Lebanon; Qatar University, QATAR

## Abstract

Hepatitis E virus (HEV) is one of the leading acute liver infections triggered by viral hepatitis. Patients infected with HEV usually recover and the annual death rate is negligible. Currently, there is no HEV licensed vaccine available globally. This study was carried out to design a multi-epitope HEV peptide-based vaccine by retrieving already experimentally validated epitopes from ViPR database leading to epitope prioritization. Epitopes selected as potential vaccine candidates were non-allergen, immunogenic, soluble, non-toxic and IFN gamma positive. The epitopes were linked together by AAY linkers and the linker EAAAK was used to join adjuvant with epitopes. Toll-like receptor (TLR)-4 agonist was used as an adjuvant to boost efficacy of the vaccine. Furthermore, codon optimization followed by disulfide engineering was performed to analyse the designed vaccine’s structural stability. Computational modeling of the immune simulation was done to examine the immune response against the vaccine. The designed vaccine construct was docked with TLR-3 receptor for their interactions and then subjected to molecular dynamic simulations. The vaccine model was examined computationally towards the capability of inducing immune responses which showed the induction of both humoral and cell mediated immunity. Taken together, our study suggests an *In-silico* designed HEV based multi-epitope peptide-based vaccine (MEPV) that needs to be examined in the wet lab-based data that can help to develop a potential vaccine against HEV.

## 1. Introduction: HEV infection

Viral hepatitis is typically known as liver infection and results in liver damage. HEV is also associated with the onset of liver inflammation [1]

HEV infections can be symptomatic as well as asymptomatic and associated with the general symptoms such as nausea, fatigue, and jaundice [2]. HEV has eight different genotypes. However, humans are vulnerable to get infected with the genotypes 1–4 [3]. Infection due to genotype 1 and genotype 2 are confined to humans and transmission is like to be done through contaminated water bodies leading to acute infection. HEV genotype 3 and 4 are considered zoonotic, and exhibit a broad host range including human, cattle, swine etc., leading towards acute infections [4]. A significant contributing factor towards this disease spread is the consumption of inadequately cooked meat [5]

Globally, the morbidity rate of HEV is around 20 million cases annually, out of which 3.3 million are symptomatic infections. However, this viral infection is associated with approximately 70,000 deaths. HEV is mostly prevalent in areas with poor sanitation and hygiene conditions [6]. HEV infections exhibit higher prevalence in European countries, while they are categorized as emerging infections in Japan and Korea. Additionally, cases of chronic HEV infection or re-infection have been documented among individuals with compromised immune systems [7].

HEV infection often goes undiagnosed and untreated, as its causes remain ambiguous and overlap with other viral hepatic infections. The diagnosis of HEV infection is confirmed by detecting anti-HEV antibodies or by utilizing an alternative approach such as qRT-PCR on plasma, stool, and serum samples [8].

Currently, there is no commercially available vaccine against HEV, therefore, the goal of this study is to design an effective vaccine against this viral infection. Traditional vaccine designing can be challenging due to the complex nature of pathogens, time-consuming and lots of experimental trial-and-error process involved in identifying suitable antigens. Henceforth, immunoinformatics, a field that integrates bioinformatics and immunology, emerge as an effective tool towards translational aspect. Immunoinformatics utilizes computational tools and algorithms to analyze large datasets and predict immunogenic epitopes, improving the efficiency and accuracy of vaccine design. By exploring the power of computational methods, immunoinformatics enables researchers to identify potential vaccine targets, optimize antigen selection, and predict vaccine efficacy. It offers a faster and more cost-effective approach to vaccine development, complementing traditional methods and accelerating the discovery of novel vaccines [9].

In this study experimentally validated epitopes of HEV were retrieved and prioritized, followed by employing immunoinformatics based tools towards the MEPV design construct. The constructed MEPV was then modelled and refined using the molecular docking technique to investigate immune responses.

## 2. Methodology

**Fig 1:**
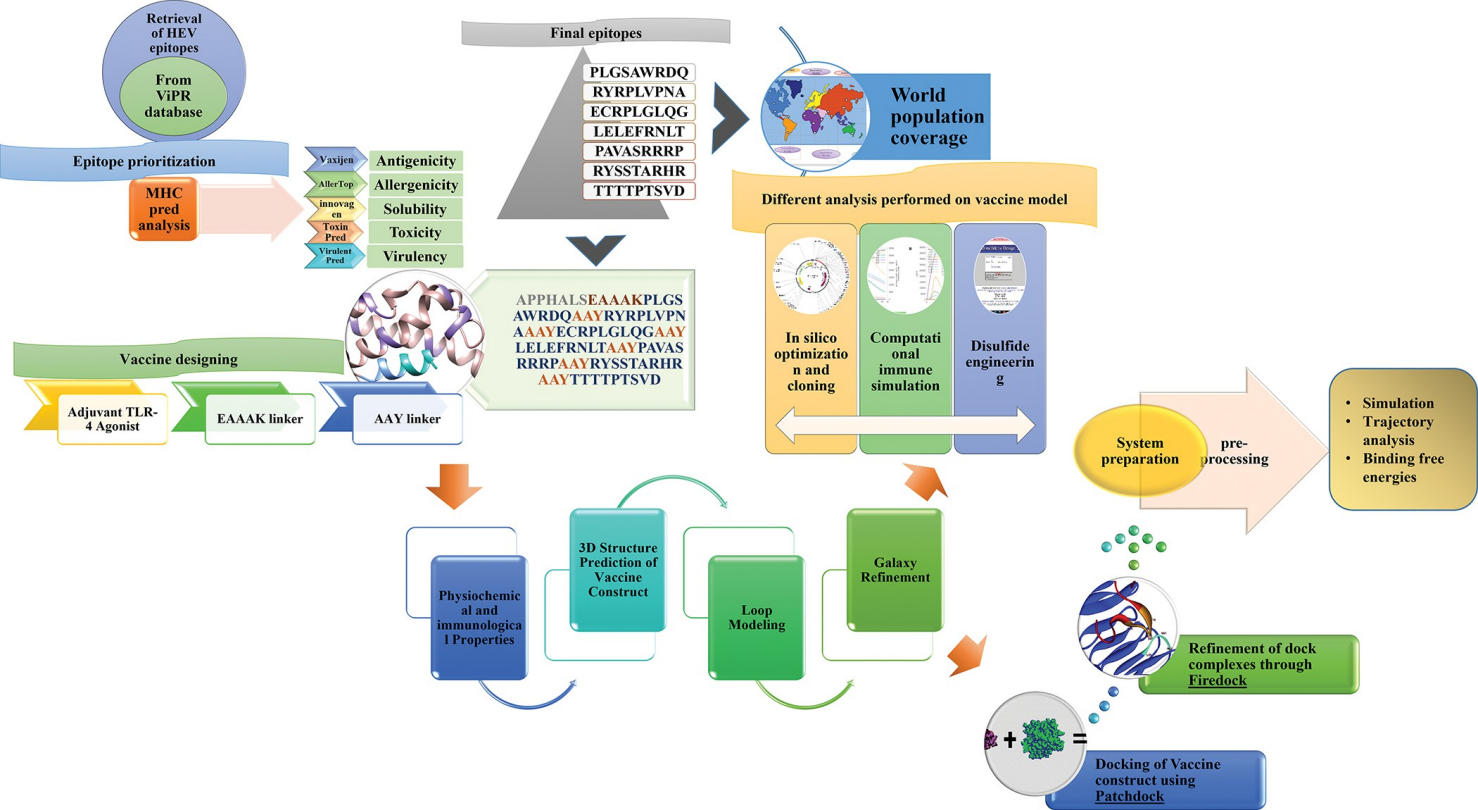
Schematic representation of the study. Flow chart of the strategy used to design a Multiepitope peptide-based vaccine against the HEV using I*n-Silico* approach.

The flowchart for this research study is shown in the Fig 1. Experimentally validated epitopes were retrieved from the database and subjected to epitope prioritization using various tools. Shortlisted epitopes being potential candidates for the vaccine construct were analysed against the set of alleles evaluating their population coverage globally. The vaccine construct based on epitopes linkers and adjuvant was modelled into a 3D structure following the loop modeling, Figure and refinement. The designed vaccine was subjected to analyses using *In-silico* cloning disulfide engineering and computational simulation before docking. To predict the interaction between the vaccine and the human body receptors molecular docking was performed followed by Molecular dynamic simulation as shown in Fig 1.

### Epitope retrieval

The Experimentally validated epitopes of HEV belonging to the species *orthohepevirus* A were retrieved from the database ViPR (viral pathogen and database and analysis resource) [10]. ViPR is an online tool that helps the researchers to search, analyze, and visualize data about different families of human pathogenic viruses. It’s user-friendly as well as reduces time. Using MHC pred (a tool to predict binding affinity for major histocompatibility complex (MHC) best possible epitopes were screened^19^. Vaxijen [11] an online tool was employed to check the antigenicity (antigenic and non-antigenic character) of the epitopes and antigenic epitopes having a threshold above 0.7 are fed into AllerTop, a web tool to inquire about the Allergenicity [12] (Allergen/non-allergen). Non-allergen epitopes were subjected to solubility check via Innovajen and soluble were further analysed for toxicity using ToxinPred [13]. Epitopes being non-toxin were examined for IFNgamma positive by the IFNepitope tool [14], followed by the Virulency evaluation on Virulent pred [15]. The population coverage for each shortlisted epitope was obtained using the Immune epitope database (IEDB)’s population coverage analysis [16].

### Designing vaccine construct

MEPVs are effective solution for treating viral infections. The vaccine construct was designed using adjuvant which is supposed to boost up the effectivity making the vaccine more immunogenic. The adjuvant TLR-4 agonist was used and connected with epitopes via linker EAAAK. AAY linkers are used for epitope-epitope junction.

### Physiological and immunological properties and 3D modeling

The vaccine construct was further subjected to allergenicity and antigenicity scans/checks using AllerTop and VaxiJen respectively. Protparam of ExPasy server [17] (tool involved in computing the physical and chemical parameters of the protein) was utilized to examine physiological properties for the earlier designed vaccine construct and then fed onto I-TASSER [18] as input sequence for 3D modeling of the vaccine construct.

### Codon optimization and disulfide engineering

An *In-silico* analysis for the stability of the structure, known as disulfide engineering, was applied using Disulfide by design 2.0 [19] associated with the phenomenon of introducing disulfide bonds in the structure. Disulfide bonds play a crucial role in stabilizing the 3D structure of mature proteins and can also have important redox activity. Disulfide engineering focuses on the formation and stabilization of disulfide bonds in proteins. By strategically introducing or modifying these bonds, the vaccine’s protein structure becomes more stable, leading to improved functionality and durability. Jcat [20] (codon adaptation tool) was used for codon optimization and adaptation. Codon optimization is a technique used to enhance the codon composition of a recombinant gene without changing the amino acid sequence. By considering different criteria, such as codon usage bias, the gene’s performance can be improved. This is feasible because multiple codons can encode the same amino acid. Codon optimization involves optimizing the genetic code of a vaccine to improve its efficiency in protein production. This optimization ensures that the vaccine’s genes are tailored to the host organism, enhancing protein expression and overall vaccine efficacy. Codon optimization was evaluated based on the percentage of the GC content and CAI (codon adaptation index) value. The CAI is a handy measure of codon usage bias. It uses a set of highly expressed genes as a reference to evaluate the effectiveness of each codon. By calculating the frequency of codon usage in a gene, a score is generated to assess its codon adaptation. Both codon optimization and disulfide engineering are essential techniques that contribute to the overall stability and effectiveness of vaccines. Jcat optimization is followed by insertion of DNA sequence of the vaccine construct in expression vector Pet-28a (+) via snapgene (software for *in-silico* cloning).

### Molecular docking

The molecular docking was performed for modelled vaccine with an immune receptor using the PatchDock server [21] (designed algorithm for molecular docking). Molecular docking plays a significant role in computational vaccine design. It helps us understand how the vaccine interacts with specific target molecules, such as viral proteins or immune receptors. By simulating the docking process, we can predict the binding affinity and stability of the vaccine-target complex. This information guides the selection and optimization of vaccine candidates, leading to more effective and targeted vaccine designs.

The vaccine model obtained was docked with the TLR-3. TLR-induced proinflammatory responses serve as the initial defence mechanism of the host. They not only combat pathogens but also facilitate the healing process to restore immune homeostasis. The molecular docking was performed with TLR3 to understand their interaction and potential immune response. TLR3, or Toll-like receptor 3, is a key component of the innate immune system. It recognizes viral RNA and triggers an immune response, leading to the production of cytokines and activation of immune cells. By docking the vaccine with TLR3, researchers can evaluate how the vaccine stimulates the immune system and potentially enhances the immune response against HEV. Results of docking via PatchDock were further refined by FireDock server [22] (web server providing high-throughput refinement) and UCSF Chimera (a program to analyse and visualize the molecular structures) was utilized for visualization.

### C-immune simulation

An in-silico simulation against the designed vaccine using C-ImmSim (an immune system simulator to investigate immunological processes) was performed to inquire probability of vaccine construct, capable of inducing immunogenicity. Epitope-immune interaction was predicted and analysed by this server, it is a computer model of cellular interactions in the immune system can mimic how immune cells communicate and respond to pathogens, aiding in understanding and improving immune function [23].

### Molecular dynamic simulation

Molecular dynamics simulation is a powerful computational technique used to study the movement and behaviour of atoms and molecules over time. It plays a crucial role in understanding the dynamics and interactions of biological molecules, such as proteins and small molecules. By simulating their motions, we can gain insights into their structural changes, binding events, and functional mechanisms. This information is valuable for drug discovery, protein engineering, and understanding biological processes at the molecular level. Molecular dynamic simulation is comprised of 3 steps including system preparation, pre-processing and simulation. Assisted Model Building with Energy Refinement (AMBER18) a software to investigate biomolecular interactions, has been employed to perform molecular dynamic simulations at 100-ns. The process of molecular dynamic simulation starts with first step of system preparation uses Antechamber module to construct the initial complex libraries. The docked complex (receptor-vaccine) was dissolved in Transferable Intermolecular Potential with 3 Points (TIP3P) water box with a border size 12. Force fields, namely "ff14SB", were employed to parameterize the receptor molecules as well as the vaccine molecules, respectively. To make the system neutralized, the procedure was followed by the inclusion of 12 NA^+^ ions. The second step of pre-processing is followed by the minimization of number of hydrogen atoms for 500 cycles and water box for 100 rounds with energy limit of 200Kcal/mol. While the alpha carbon atoms were minimized with energy limit of 5Kcal/mol for 1000 rounds, and non-heavy atoms were minimized for 300 runs with 100Kcal/mol energy limit on the remainder of the system. Following that, the systems were given the temperature of 300k for 20ps in the Number of particles Volume and Temperature (NVT) ensemble under the conditions that were periodically bounded, while hydrogen bond atoms were controlled by spherical harmonic accelerated kinetic energy (SHAKE) algorithm. The system was equilibrated for 100 ps. In addition, pressure was equilibrated in Number of particle Pressure and Temperature (NPT) ensemble (used to determine the equation of state) for 50ps, initially with restrain check on carbon atoms and then without any restriction. System equilibration was completed after the preceding procedures were completed. The preset cut-off value for unbounded interactions was set at 0.8, and the run for production was conducted for 100ns. To conduct trajectories, the Conformational Analysis by Protein Packing Trajectories (CPPTRAJ) command was used. The resulting trajectories were visualised by visual molecular dynamics (VMD) to detect the interaction between the vaccine and the receptors of human immune system.

## 3. Results

### Retrieval and prioritization of potential epitopes

Experimentally determined epitopes of HEV retrieved from ViPR, were used to design vaccine construct against HEV. Potential epitope screening was done while keeping different parameters including antigenicity, allergenicity, solubility, toxicity, IFN-gamma inducing, and virulence. Experimentally validated epitopes retrieved from VIPR database were 208 in number and were fed into the MHC pred server. Those having there IC50 < 100 were selected for further analysis, of those which were allergens, poorly soluble, non-antigen, toxins, and IFN-gamma negative were discarded. The 7 shortlisted epitopes were ligated with linkers to design a vaccine construct. The threshold set for antigenicity was 0.6 and the antigenicity value for these 7 epitopes were ranging from 0.7–2.6 and virulency was 0.98 for all except PLGSAWRDQ having a virulency score of 1.05. All these epitopes were used for MEPV construct designing (Table 1).

**Table 1:** Selected epitopes to be used to design multi-epitope vaccine construct.

Epitope Sequence	MHC PRED	IC50	Allegenicity	Antigenecity	Antigenecity score	Solubility	Toxicity	IFN gamma	Virulency	virulency score
AGPRVRQPARPLGSAWRDQAQRPAV	PLGSAWRDQ	40.46	Non-Allergen	Antigen	0.8205	soluble	Non-Toxin	Positive	Virulent	1.0595
ARATIRYRPLVPNAVGGYAISISFW	RYRPLVPNA	2.33	Non-Allergen	Antigen	0.7326	soluble	Non-Toxin	Positive	Virulent	0.9892
CPECRPLGLQGCAFQSTVAELQRLK	ECRPLGLQG	46.88	Non-Allergen	Antigen	1.6348	soluble	Non-Toxin	Positive	Virulent	0.9892
DFALELEFRNLTPGNTNTRVSRYSS	LELEFRNLT	35.48	Non-Allergen	Antigen	2.6224	soluble	Non-Toxin	Positive	Virulent	0.9892
DQAQRPAVASRRRPTTAG	PAVASRRRP	5.61	Non-Allergen	Antigen	1.9281	soluble	Non-Toxin	Positive	Virulent	0.9892
RNLTPGNTNTRVSRYSSTARHRLRR	RYSSTARHR	1.17	Non-Allergen	Antigen	1.0103	soluble	Non-Toxin	Positive	Virulent	0.9892
SISFWPQTTTTPTSVDMNSITSTDV	TTTTPTSVD	13.24	Non-Allergen	Antigen	0.9578	soluble	Non-Toxin	Positive	Virulent	0.9892

### Population coverage analysis

**Fig 2:**
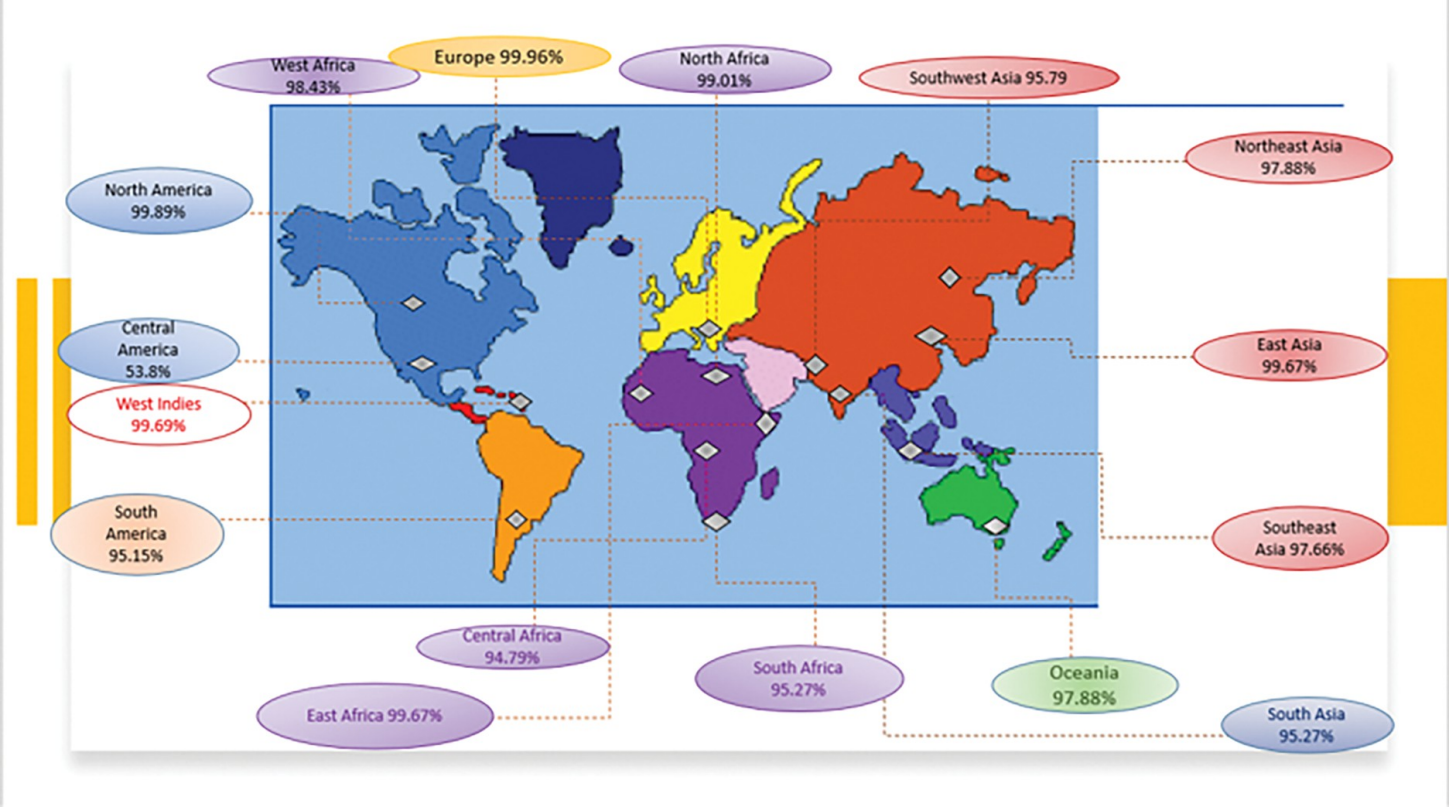
Population coverage analysis. Schematic representation of global population coverage of the selected epitopes.

**Fig 3:**
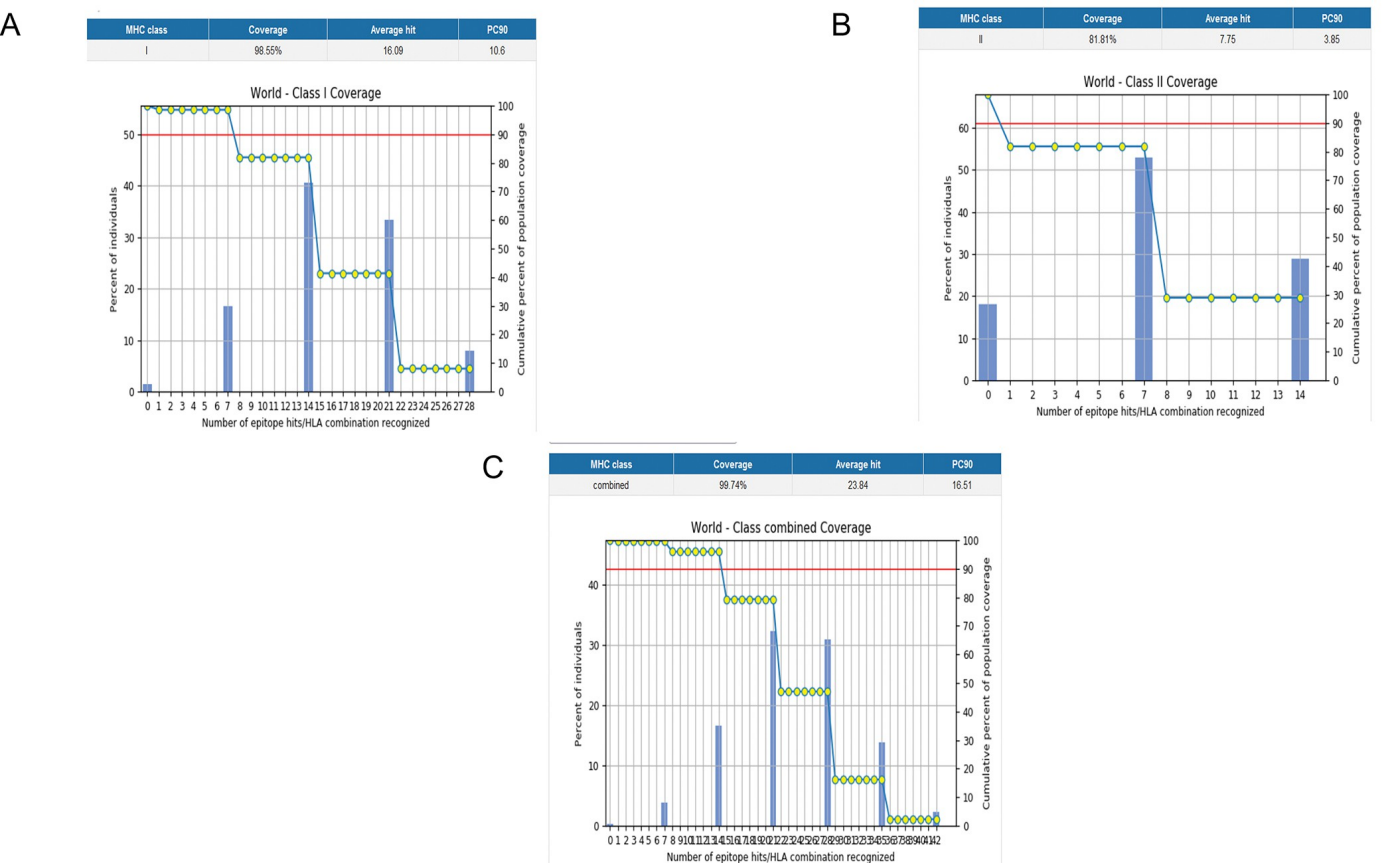
MHC Class-wise population coverage of the prioritized epitopes. A) World-class MHC I population coverage. B) World-class MHC II population coverage. C) Class-combined global population coverage graph.

Global population coverage analysed by IEDB’s population coverage analysis server. The epitopes interacting with the set of targeted alleles were covering 99.74% population globally. Population coverage from different geographic regions was analysed as shown in Fig 2. The world population coverage for MHC I was found to be 98.55% depicted in Fig 3A and 81.81% for MHC II as shown in Fig 3B. While class-combined population coverage was 99.74% (Fig 3C). The predicted PC50 value for MHC I was 10.6 and for MHC II was 3.85. High population coverage is desirable because it means a larger portion of the population is vaccinated, which can contribute to the potential effectiveness of a vaccine. When a significant percentage of the population is immunized, it creates a collective immunity known as herd immunity. This helps protect individuals who are unable to receive the vaccine due to medical reasons or have a weakened immune system. Additionally, high population coverage reduces the overall transmission of the disease, making it harder for the pathogen to spread and infect susceptible individuals. This ultimately helps control the spread of infectious diseases and reduces the likelihood of outbreaks [Figs 2 and 3].

### Vaccine construct

**Fig 4:**
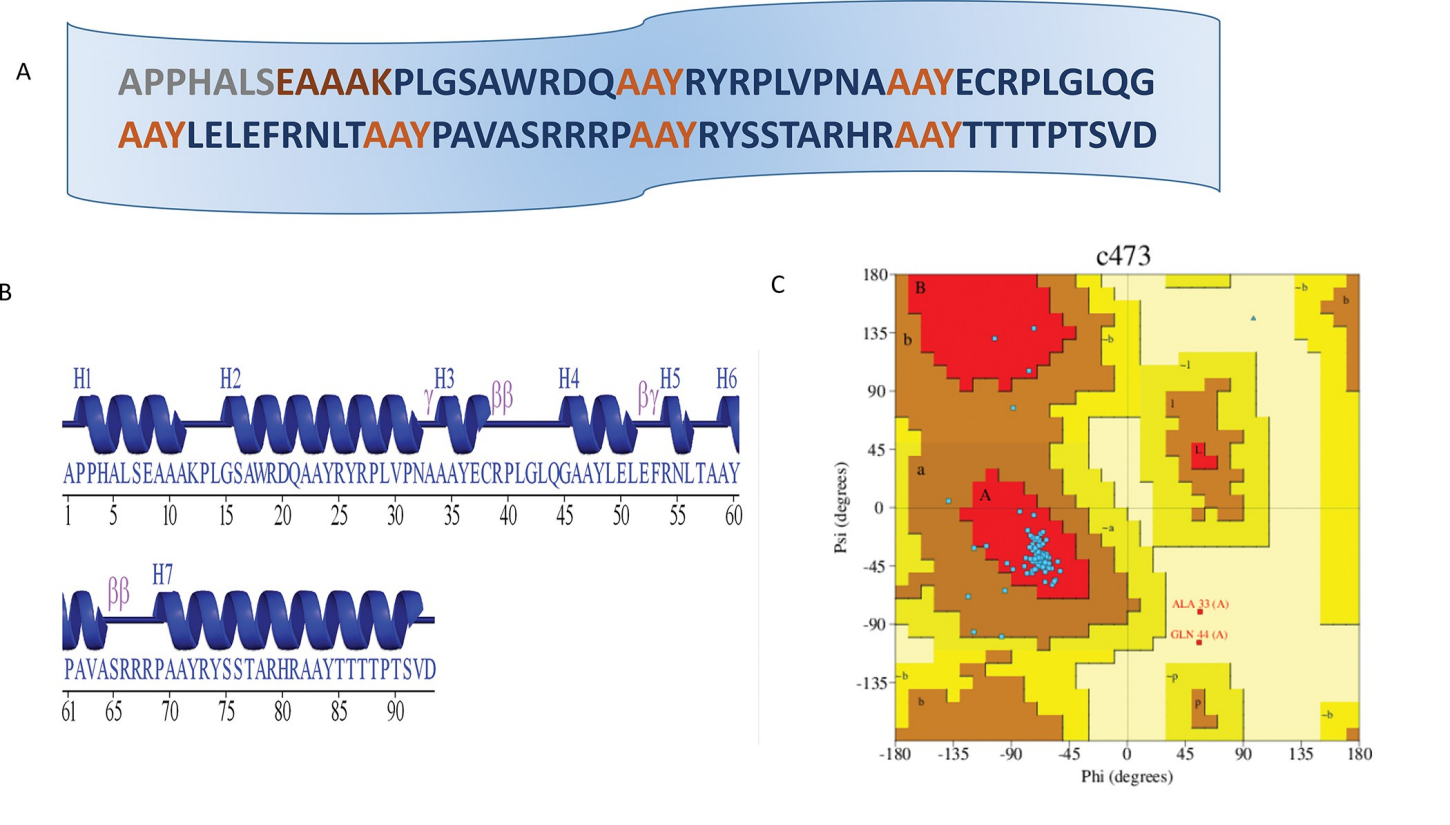
Characteristics of designed vaccine construct. A) Designed vaccine construct consisting of epitopes, adjuvants, and linkers. B) The predicted secondary structure of vaccine sequence. C) The Ramachandran chart depicting 86% residues in its most favoured region.

Vaccines designed from epitopes using a computational approach are reliable and can be more beneficial because they can be produced by using simple computational tools and provide specific immune responses. Shortlisted 7 epitopes were linked by AAY (linker) in order to design a MEPV construct. EAAAK linker is used to link TLR-4 Agonist to MEPV construct to increase immunogenicity so that immune response can be enhanced against the antigen. The resulting vaccine construct was comprised of 93 amino acids as shown in Fig 4.

Fig 4A is the depiction of vaccine’s amino acid sequence. The amino acids in favoured regions of the vaccine model are 86%, 8.9% in the additional allowed region while the Ramachandran’s disallowed region was showing 2.5% amino acids. (Fig 4C). Secondary structure predicted for the vaccine model was having 7 helices, 12 helix-helix interaction, 5 beta-turns and 2 gamma-turns, as in Fig 4B.

### Multiepitope peptide-based vaccine

**Fig 5:**
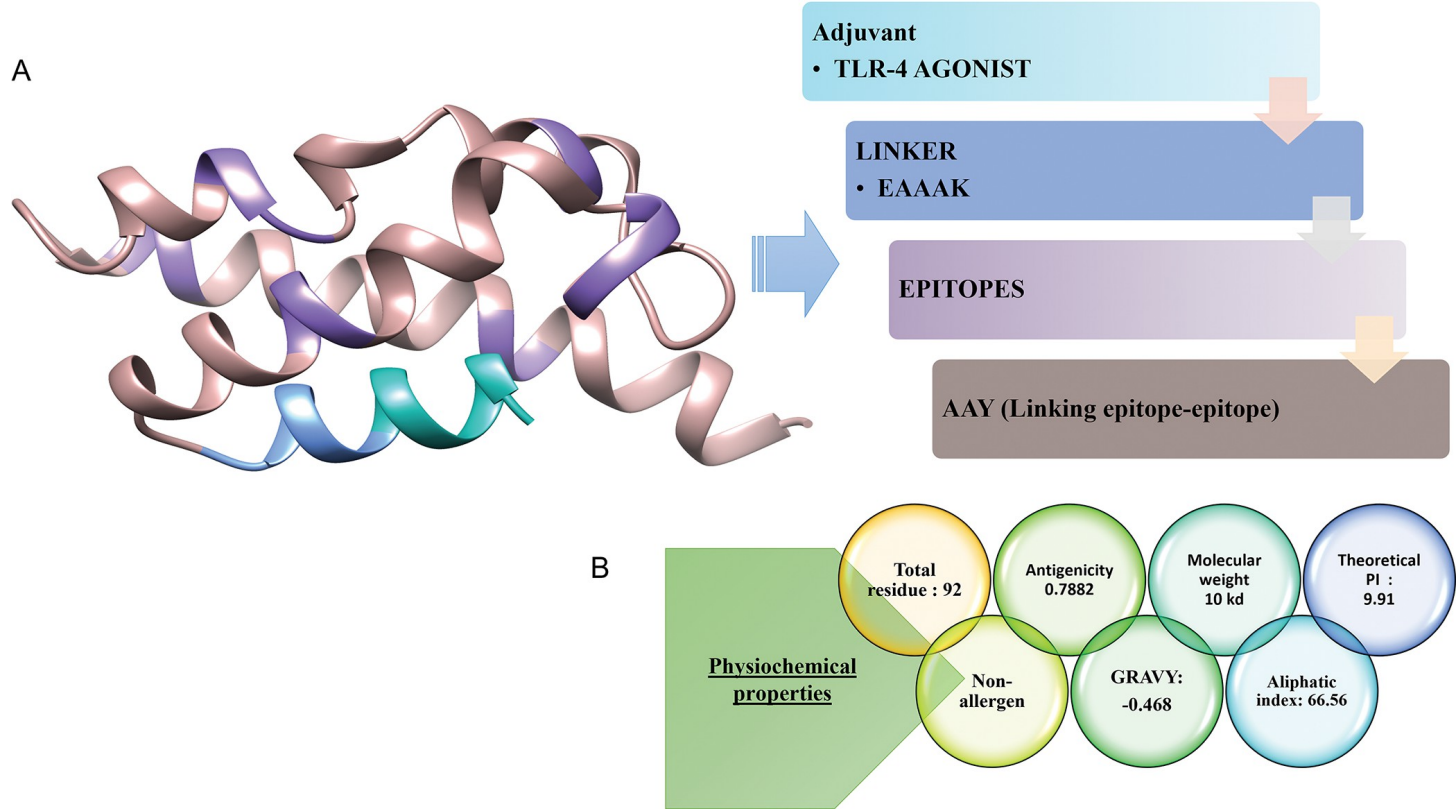
Structural characteristics of the vaccine model. A) Three-dimensional model for multi-epitope vaccine model. B) physiochemical properties of the vaccine model.

A MEPV model was obtained using I-tasser first model based on Z- score was selected and then subjected to loop modeling. Loop modeling of the 5 loops of the predicted model was done using galaxy web and then sent for refinement. The 3D structure for the vaccine model is given in Fig 5A. Binding energies for the top 10 models are shown in Table 2. Model 1 was selected using the criteria of low Molprobity, low galaxy energy and high RMSD value. The values for RMSD, Molprobity ang Galaxy energy were 2.295, 1.541, and -1794 (the lowest energy score favours the best model) respectively.

**Table 2:** Binding energy details for the galaxy refined models of the multi-epitope peptides-based vaccine.

Model	RMSD	Mol Probity	Clash score	Poor rotamers	Rama favoured	GALAXY energy
Initial	0	3.025	13.6	8.8	84.6	-684.31
MODEL 1	2.295	1.541	3.2	0	93.4	-1794
MODEL 2	1.413	1.386	1.9	0	93.4	-1790.5
MODEL 3	1.747	1.164	1.9	0	96.7	-1790.1
MODEL 4	1.73	1.431	1.9	0	92.3	-1772.6
MODEL 5	1.209	1.486	3.2	0	94.5	-1771.9
MODEL 6	1.626	1.515	2.6	0	92.3	-1771.8
MODEL 7	0.742	1.486	3.2	0	94.5	-1770.4
MODEL 8	1.548	1.553	2.6	0	91.2	-1769.9
MODEL 9	1.181	1.226	1.3	0	94.5	-1769.2
MODEL 10	1.792	1.332	1.9	0	94.5	-1768.2

MEPV construct after being designed were examined under different physiological and biochemical properties (Fig 5B). Total number of residues are 93, GRAVY was -0.468. GRAVY value is calculated to determine hydrophobicity and hydrophilicity for the sequence, while the value being negative depicts that the designed construct was having hydrophilic nature. Being non-allergen and having antigenicity of 0.788 the construct was considered overall stable.

### Disulfide engineering

**Fig 6:**
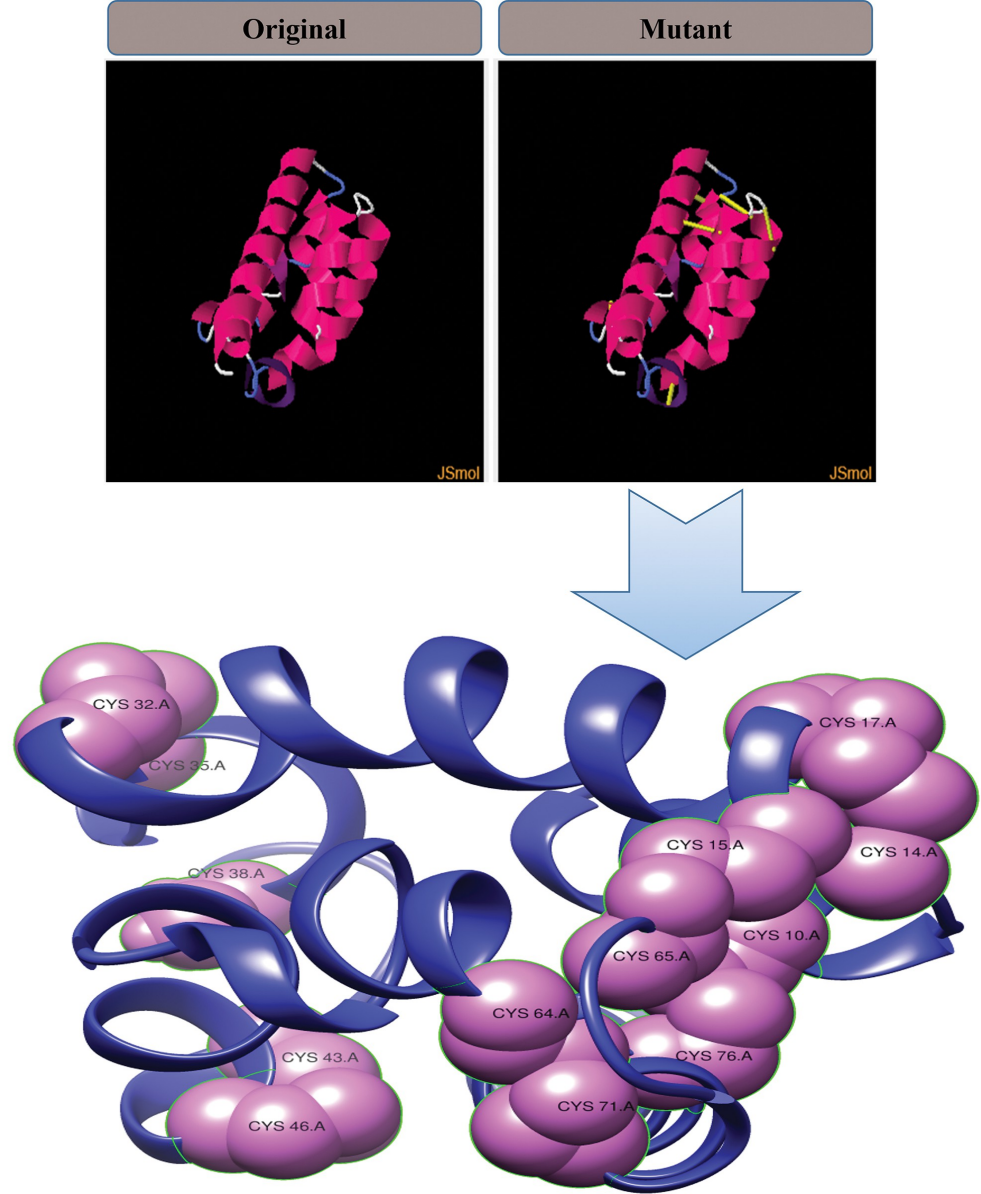
Mutant model for vaccine generated by introducing cysteine residues (shown in violet).

The designed vaccine was gone through the process of disulfide engineering that can provide stability, acquiring a geometric conformation. A mutant to the vaccine was obtained and residues pairs having unfavourable interactions energy regarding vaccine stability were mutated as cysteine residues. Mutated residues have a binding energy > 1 kcal/mol. For this designed vaccine 6 residue pairs were mutated. All 6 residues with angle and binding energy are given in Table 3. The mutant vaccine model with its mutated cysteine residues is shown in Fig 6.

**Table 3:** Pairs of residues mutated as cysteine.

Residue 1	Residue 2	X3	Kcal/mol
10 ALA	76 SER	-109.9	5.88
14 LEU	17 ALA	112.16	5.26
15 GLY	65 SER	-77.62	4.5
32 ASN	35 ALA	99.26	6.3
43 LEU	46 ALA	95.97	4.47
64 ALA	71 ALA	85.91	5.44

### 3.6 *In- silico* cloning

**Fig 7:**
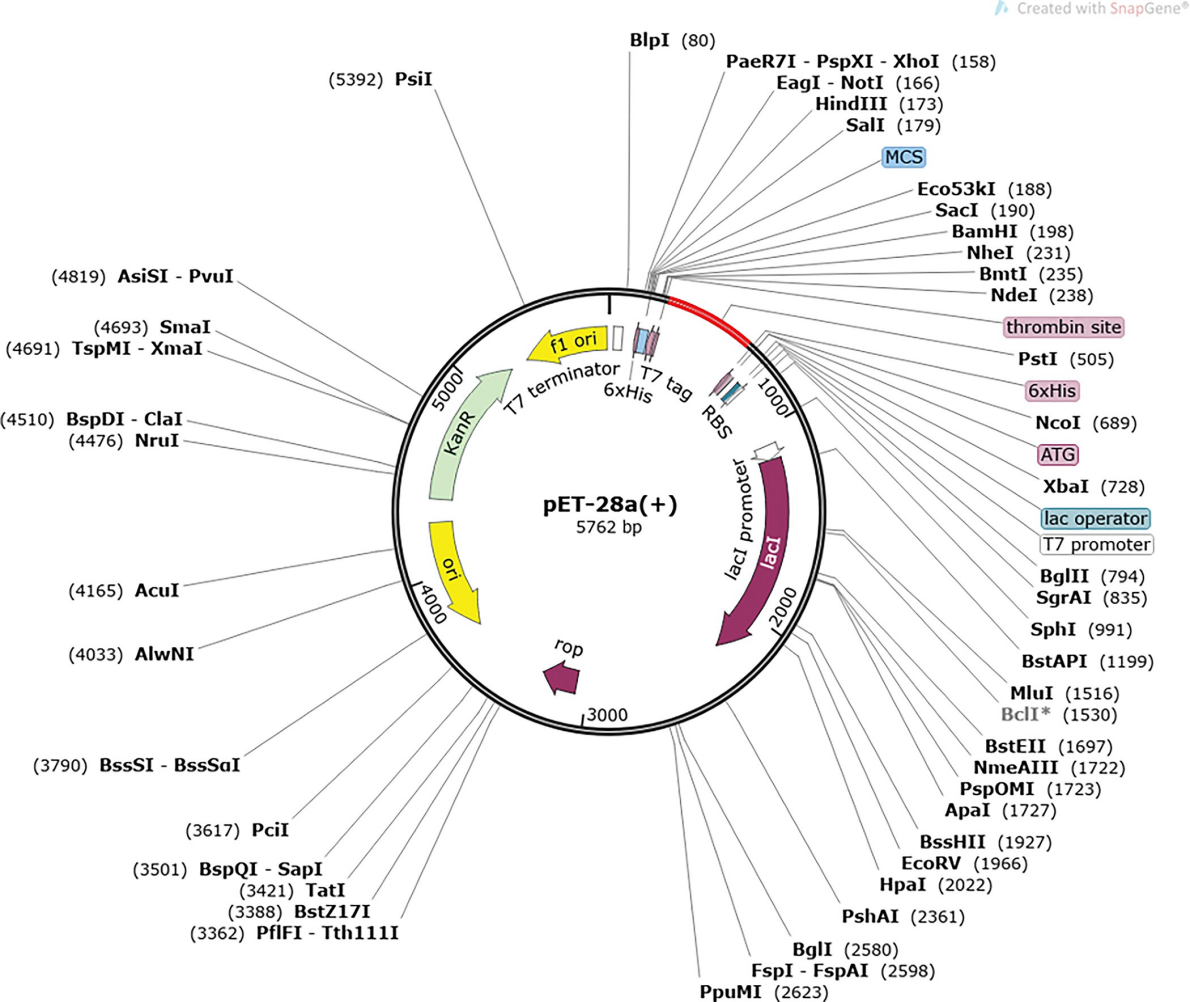
In-silico cloning using E. coli expression system. Reverse translated vaccine’s DNA sequence as shown in red, expressed in pET-28a (+) vector. GC content for the designed vaccine was 56.23% while the CAI value was 1.0.

Using J-Cat server the vaccine sequence was processed into a DNA sequence being reversely translated acquiring a high level of expression in standard organism *E*. *coli*. For recombinant proteins, the expression system (*E*. *coli*) was specified, followed by codon optimization assuring the production of recombinant vaccine proteins at a higher level in the *E*. *coli* k12 system (Fig 7).

#### Molecular docking

**Fig 8:**
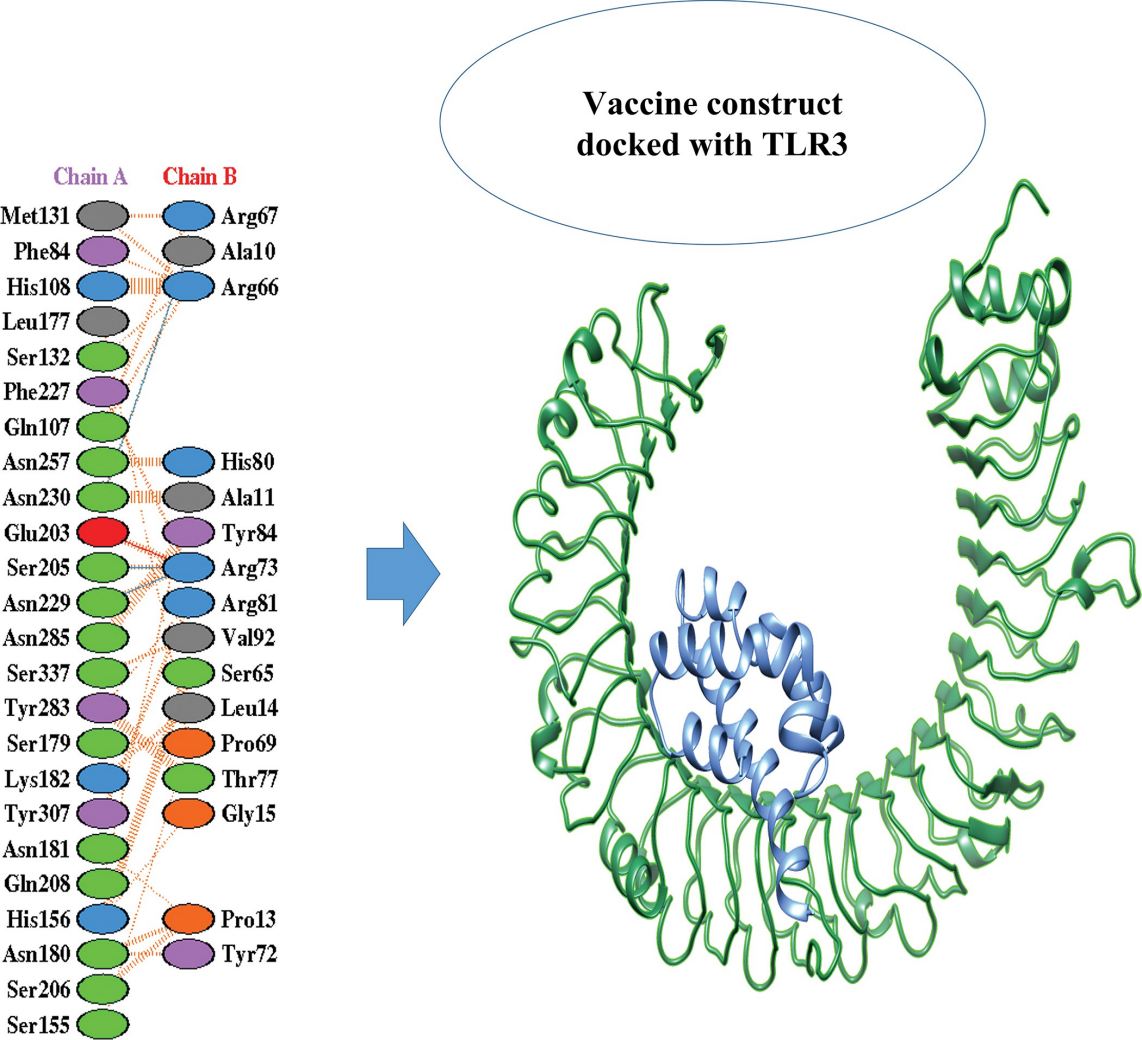
Multiepitope peptide HEV vaccine docked with the human immune receptor. TLR3-complex and protein-protein interaction between the chains.

A computational method (molecular docking) is performed to examine interactivity between the vaccine model and human immune receptors leading an idea towards drug/vaccine development [24]. So, the immune receptor (TLR-3) was selected to predict the binding interactions. Blind docking (for an unknown target site) [25] was applied using patchdock. The designed vaccine construct was docked with TLR-3 receptor. TLR3 (toll-like receptor 3) is considered as a PPR (pattern recognition receptor) and is supposed to be an inducer for type 1 interferon development, playing a key role in activating both types of immune responses [26]. Patchdock top solutions for all the receptors were submitted to Firedock (online server) for refinement of predicted dock complexes and top 10 refined solutions were obtained. The solution ranking is based on the global energies. For TLR3 docked complex solution 1 having -35.18 (Table 4) global energy was selected. The dock complex for TLR-3 and their Protein-Protein interaction obtained from PDBSum are shown in Fig 8.

**Table 4:** Energies calculated for TLR-3-complex.

Ranks	Solution Number	Global Energy	Attractive VdW	Repulsive VdW	ACE	Hydrogen bond
1	1	-35.18	-28.88	5.26	9.60	-3.18
2	9	-22.45	-29.78	19.59	16.64	-8.61
3	8	-18.62	-27.62	8.68	12.10	-2.44
4	4	-16.10	-29.54	11.53	14.69	-7.20
5	10	-11.60	-18.93	3.73	6.02	-0.24
6	6	-3.78	-22.53	9.84	10.08	-2.71
7	2	3.33	-30.74	11.48	15.00	-5.00
8	3	9.79	-6.01	0.94	2.55	-1.28
9	7	12.82	-22.17	28.13	5.99	-1.70
10	5	39.71	-19.76	13.36	11.88	-1.51

Salts bridges between both protein chains of the dock complexes are shown in red while hydrogen bonds are coloured blue, dilsufide bonds are yellow while, doted orange lines are unbound interactions.

### Computational modeling of immune simulation

**Fig 9:**
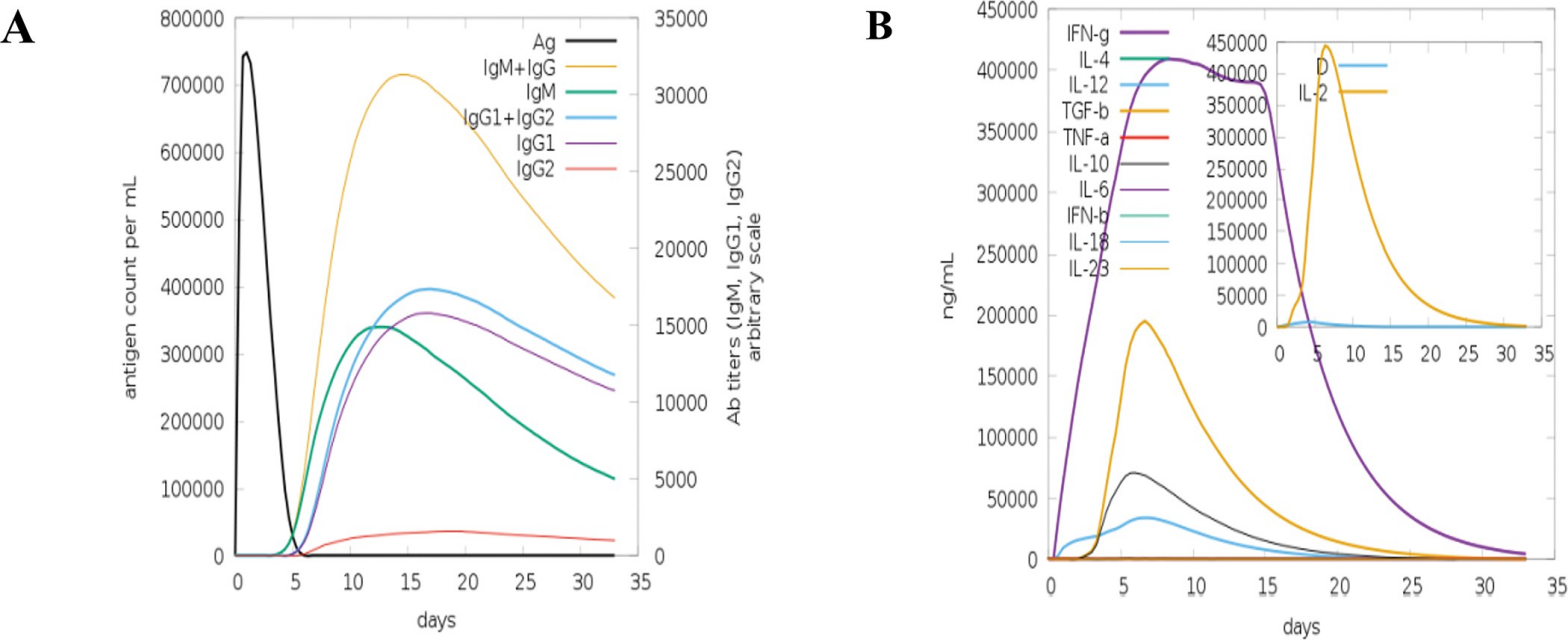
Computationally immune simulated host immune response towards Multiepitope peptide HEV vaccine of A) Production of immunoglobulins in response to vaccine model. B) Induced interleukins in response to vaccine model The purpose of Computational immune simulation was to get an immunogenic profile for the designed vaccine [27]. The designed vaccine was observed as inducing primary and secondary immune responses. The production of IgM+IgG was observed at a higher level leading to the production of IgG1 and IgG2 depicting the induction of primary immune response. Production of IgM+IgG was raised up to 700000/ml in the first 15 days, then there is a decline as shown in Fig 9A. IgM and IgG are types of antibodies produced by the immune system in response to an infection or vaccination. It was observed that IFN-g induced was in greater amount while the release of IL-also happened up to 10 days and then start decreasing. IL stands for interleukin, which is a group of cytokines involved in regulating immune responses and inflammation. IFN-g was produced at the higher rate. Its production reached 400000/ml in 5 days and the decrease in its production can be seen after 20 days (Fig 9B). IFN-g refers to interferon-gamma, a type of cytokine that plays a role in immune responses against viral and bacterial infections.

C-IMMSIM server was used for immune simulation as shown in Fig 9.

### Molecular dynamic simulations

**Fig 10:**
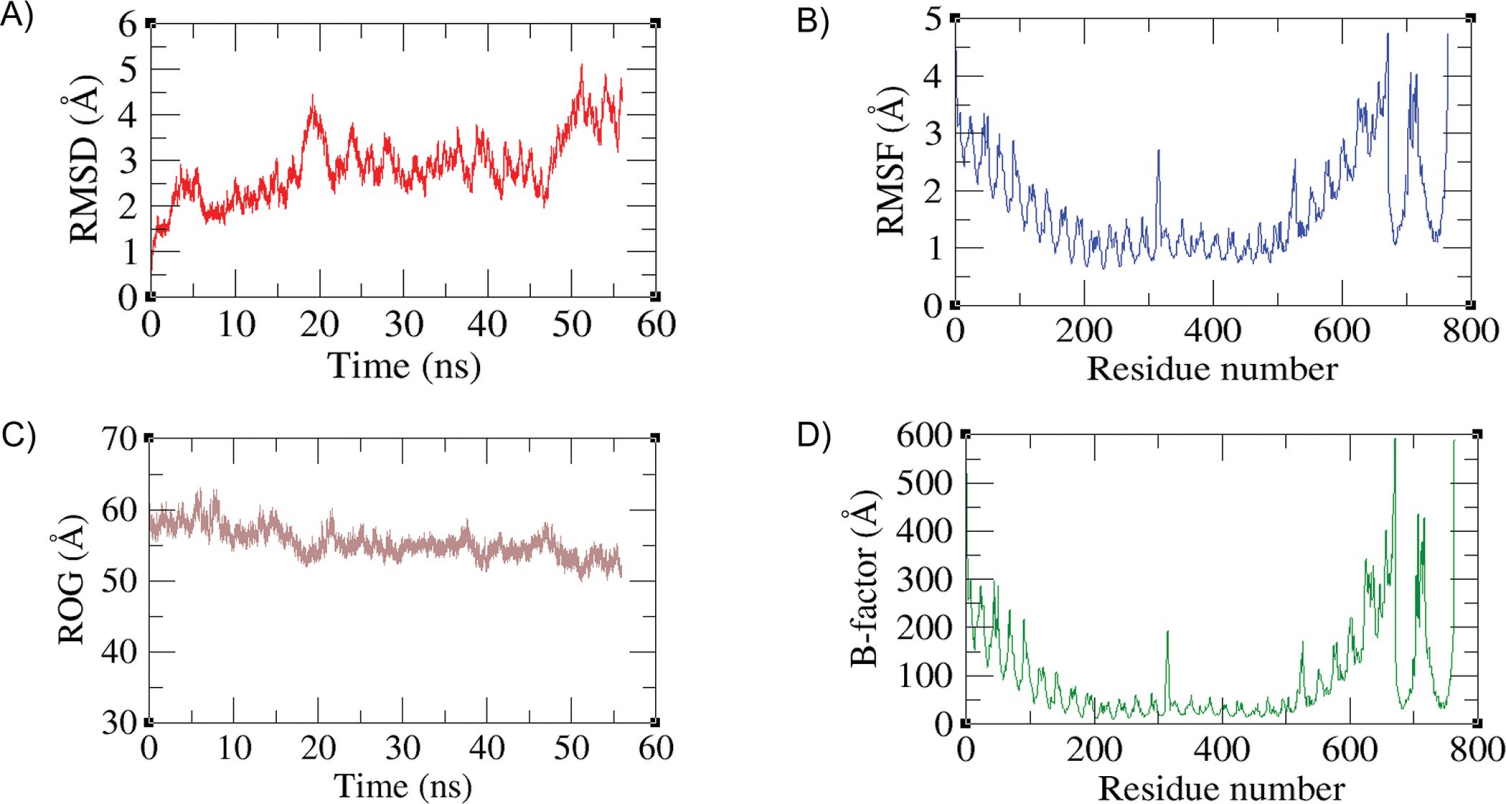
Trajectories obtained for simulated complexes (vaccine and receptor). A) RMSD graph. B) RMSF graph. C) ROG graph. D) B-factor graph.

Molecular dynamic simulations were employed on vaccine-TLR-3 complex. The resulting trajectories were based on four parameters including root mean square deviation (RMSD), root mean square fluctuations (RMSF), radius of gyration (ROG) and *β*-factor (Temperature Factor). The obtained values for terms like RMSD, RMSF, RoG, and *β*-factor provide information about the structural characteristics and dynamics of biomolecules. Graphical representation for all these parameters has been shown in Fig 10.

RMSD was calculated to interpret the structure stability for vaccine-TLR-3 complex. RMSD measures the average deviation between the positions of atoms in a molecule compared to a reference structure. The average RMSD calculated for the system was 3.5Å while the maximum was 5.1 Å at 50ns with few variations (Fig 10A). RMSF indicates the flexibility or fluctuation of atoms within a molecule. The residue fluctuation was observed as 1.4 Å with maximum residue fluctuation of 4.5 Å (Fig 10B) depicting the stability of binding modes of the vaccine was disturbed. Protein compactness and relaxation is predicted by the estimation of ROG. RoG represents the compactness or size of a molecule. The ROG estimated was 55Å (Fig 10C) and remained stable confirming the stability of the protein. B-factor is the estimation of the residual deviation based on temperature. B-factor reflects the thermal motion or flexibility of atoms in a crystal structure. The beta-factor observed as 50Å with few variations the maximum Beta-factor obtained as 590 Å (Fig 10D) showed that binding mode of vaccine with receptor cell got disturbed due to temperature. These values help researchers understand the conformational changes, stability, and dynamics of biomolecules.

## 4. Discussion

MEPV against HEV has shown its effectiveness and highlighted its advantages over conventional vaccines. The fact that it is cost-effective and can be developed quickly is a significant breakthrough. Considering that there is currently no globally recognized HEV vaccine, our research provides a valuable and innovative idea to combat HEV infections, which are particularly common in Asia and many European countries. This work has the potential to make a meaningful impact in the field of HEV prevention. The computational analysis performed on MEPV indicates that it is highly effective in stimulating immune responses and triggering the necessary processes. This is a significant finding that highlights the potential of the vaccine in combating HEV infections. The need for an effective HEV vaccine arises from the significant burden of Hepatitis E virus infections worldwide. As an RNA virus, HEV can cause acute hepatitis and occasionally progress to chronic infection, leading to severe liver disease. Developing a vaccine against HEV is crucial for preventing transmission and reducing the global impact of this virus. By providing protection against HEV infection, a vaccine can contribute to public health efforts and improve overall well-being. HEV is involved in liver infection along with the extrahepatic indications and the treatment is still not specified. Vaccination is considered as the most efficient and effective method to treat infections. Thus, a vaccine against the HEV infection is required. MEPV designing using computational approaches is a very cost-effective method, take very less time and is making advancements in vaccine designing. The use of immunoinformatics and computational research techniques to design an epitope-based vaccine is better option as compared to the conventional method of vaccine designing which takes much time and show less efficiency and effectivity than the epitope-based vaccine [28]. A similar research based on the use of immunoinformatics states that this approach of vaccine designing is leading to promising results against the viral infections [29]. A MEPV made from experimentally validated epitopes of HEV can be highly useful in combating HEV infections. By incorporating these specific epitopes into the vaccine, it can effectively target the viral proteins and elicit a targeted immune response. This approach enhances the vaccine’s ability to induce a strong and specific immune response against HEV, potentially providing better protection against the virus. The use of experimentally validated epitopes ensures that the vaccine is designed based on reliable and accurate information, increasing its efficacy and safety. In this study we picked the experimentally validated epitopes of HEV from ViPR, shortlisted 7 vaccine candidate conforming the physiochemical properties to design MEPV against HEV infection. MEPVs are proficient enough to activate both types of immune responses; the cellular and the humoral proving itself better than the monovalent vaccines [30]. Experimentally validated epitopes used in our study are already validated as epitopes of HEV, so they were directly scrutinized for epitope prioritization. Epitope prioritization is the process of selecting potential epitopes and evaluating them on different parameters. Epitope were subjected to antigenicity, allergenicity, toxicity, solubility and IFNgama positive/negative checks. Epitope obtained after prioritization are observed as safer, efficient, and having potential to induce long lasting immune responses [31].

The potential immunogenic epitopes shortlisted after different checks were linked together to design the vaccine construct. TLR-4 agonist used as an adjuvant in vaccine construct designed for HEV infection. Adjuvant was linked with epitopes via EAAAK linker, while AAY linkers were used to design epitope-epitope connection. Adjuvant aid in boosting up the vaccine’s effectiveness and functionality. Linkers avoid overlapping between epitopes and infer stability into structure, also they have role in activating immune system [32]. TLR-4 agonist specifically involved in the activation of T cells used as an adjuvant and helps in raising the immunogenic property of the vaccine. It considered safe promising the efficacy and effectiveness of the vaccine [33]. Use of vaccine globally confirms its efficacy; therefore, population coverage analysis was performed. The vaccine was covering 99.74% of the world population.

Physiochemical properties of vaccine construct were evaluated. Molecular weight of 10kd and the GRAVY value obtained from Protparam was -0.463 for MEPV against HEV. Molecular weight less than 100kd and more negative GRAVY score depicts the more stable and best structure/construct [34]. 3D structure was predicted and verified by plotting and analysing the Ramachandran plot which highlights 88.86% residues in the most favoured region and 2.5% in disallowed region referring to a good quality. However, the best structure quality is depicted if 90% residues are in Rama favoured region and disallowed region of plot is marked with 0 residues [34]

The MEPV against HEV infection designed in this study were then subjected to molecular docking. HEV vaccine was docked with TLR-3 (receptor of human body) using patchdock followed by the refinement of the dock complexes by fire dock. Dock complex obtained for the TLR-3 showed a good protein-protein interaction. The Global energy prediction for the dock complex was -35.18. Docking with TLR-3 come up with the best interaction predicting the idea of activating good immune response. More negative the global energies for the dock complexes more effective the docking will be resulting into a better dock complex thought to be involved in inducing the immunity. Molecular docking is performed to predict an interaction and binding affinity among the ligand (vaccine model) and receptor (immune receptors like Toll like receptors). This interaction evaluates the efficacy of the designed vaccine that to which extent it can induce immunity and activate the immune system [35].

The MEPV was designed against HEV previously. In comparison to our study, they have used capsid protein only leading towards the epitope prediction of helper T-lymphocytes (HTL) [36]. Furthermore, they performed in vitro analysis based on the results of their *in-silico* approach. While epitopes with experimental validation were utilized in our study.

## Conclusion

HEV is a self-limiting yet acute infection prevalent in developing countries with poor hygiene and sanitation. Immunocompromised individuals more are vulnerable to chronic infections. It can be fatal to patients with other clinical manifestations. There is no proper treatment for HEV to date nor any vaccine introduced globally. Conventionally produced vaccines are still under trial, to save time we proposed idea of MEPV against HEV that is considered good in terms of efficacy, using an immunoinformatics approach and reverse vaccinology. Several analyses were performed in this study to test efficacy of the proposed /designed vaccine and ended up with satisfactory results.

Based on this study, we suggest that the proposed vaccine model is effective in terms of provoking immune responses in humans, but its efficacy can be improved by adding up or using different adjuvants. Nevertheless, these *In-silico* approaches provide promising results, however, it needs to be validated with experimental findings to draw a conclusive assessment of immunological response against HEV infection.
